# L-leucine, beta-hydroxy-beta-methylbutyric acid (HMB) and creatine monohydrate prevent myostatin-induced Akirin-1/Mighty mRNA down-regulation and myotube atrophy

**DOI:** 10.1186/1550-2783-11-38

**Published:** 2014-08-13

**Authors:** Christopher Brooks Mobley, Carlton D Fox, Brian S Ferguson, Rajesh H Amin, Vincent J Dalbo, Shawn Baier, John A Rathmacher, Jacob M Wilson, Michael D Roberts

**Affiliations:** 1 School of Kinesiology, Molecular and Applied Sciences Laboratory, Auburn University, 301 Wire Road, Office 286, Auburn, AL 36849, USA; 2 Department of Health Sciences and Human Performance, University of Tampa, Tampa, FL, USA; 3 Harrison School of Pharmacy, Auburn University, Auburn, AL, USA; 4 School of Medical and Applied Sciences, Central Queensland University, Rockhampton, QLD, Australia; 5 Metabolic Technologies, Inc, Ames, IA, USA

**Keywords:** Myostatin, GDF8, Akirin-1, Atrophy, Skeletal muscle

## Abstract

**Background:**

The purpose of this study was to examine if L-leucine (Leu), β-hydroxy-β-methylbutyrate (HMB), or creatine monohydrate (Crea) prevented potential atrophic effects of myostatin (MSTN) on differentiated C2C12 myotubes.

**Methods:**

After four days of differentiation, myotubes were treated with MSTN (10 ng/ml) for two additional days and four treatment groups were studied: 1) 3x per day 10 mM Leu, 2) 3x per day 10 mM HMB, 3) 3x per day 10 mM Crea, 4) DM only. Myotubes treated with DM without MSTN were analyzed as the control condition (DM/CTL). Following treatment, cells were analyzed for total protein, DNA content, RNA content, muscle protein synthesis (MPS, SUnSET method), and fiber diameter. Separate batch treatments were analyzed for mRNA expression patterns of myostatin-related genes (Akirin-1/Mighty, Notch-1, Ski, MyoD) as well as atrogenes (MuRF-1, and MAFbx/Atrogin-1).

**Results:**

MSTN decreased fiber diameter approximately 30% compared to DM/CTL myotubes (p < 0.001). Leu, HMB and Crea prevented MSTN-induced atrophy. MSTN did not decrease MPS levels compared to DM/CTL myotubes, but MSTN treatment decreased the mRNA expression of Akirin-1/Mighty by 27% (p < 0.001) and MyoD by 26% (p < 0.01) compared to DM/CTL myotubes. shRNA experiments confirmed that Mighty mRNA knockdown reduced myotube size, linking MSTN treatment to atrophy independent of MPS. Remarkably, MSTN + Leu and MSTN + HMB myotubes had similar Akirin-1/Mighty and MyoD mRNA levels compared to DM/CTL myotubes. Furthermore, MSTN + Crea myotubes exhibited a 36% (p < 0.05) and 86% (p < 0.001) increase in Akirin-1/Mighty mRNA compared to DM/CTL and MSTN-only treated myotubes, respectively.

**Conclusions:**

Leu, HMB and Crea may reduce MSTN-induced muscle fiber atrophy by influencing Akirin-1/Mighty mRNA expression patterns. Future studies are needed to examine if Leu, HMB and Crea independently or synergistically affect Akirin-1/Mighty expression, and how Akirin-1/Mighty expression mechanistically relates to skeletal muscle hypertrophy *in vivo*.

## Background

Myostatin (MSTN) is a key negative regulator of mature skeletal muscle myofiber growth [1-3]. In this regard, MSTN has been shown to reduce muscle protein synthesis by abrogating mTORC1 signaling [4,5] and increase muscle proteolytic mechanisms [6,7]. Furthermore, several studies have implicated physical inactivity-induced up-regulation in MSTN as a potential regulator in age-related skeletal muscle loss [8-13], and there is supporting evidence suggesting that serum and skeletal muscle MSTN are elevated with aging [14,15].

The effects of exercise have also been implicated in MSTN pathway signaling. Specifically, Louis et al. [16] reported that MSTN mRNA expression is depressed within hours following either endurance or resistance exercise. Dalbo et al. [17] similarly reported that resistance exercise decreased skeletal muscle myostatin mRNA levels up to 6 hours post-exercise. Likewise, recent evidence suggests that skeletal muscle MSTN increases three days following detraining from 90 days of resistance exercise; an event which preceded subtle but rapid type II fiber atrophy [18].

While exercise reduces skeletal muscle myostatin expression, nutritional strategies to reduce myostatin signaling are also warranted. In this regard, select nutrients have been shown to increase skeletal muscle anabolic signaling mechanisms. Leucine is a well-known activator of mTOR signaling [19-21], and leucine has additionally been shown to reduce muscle proteolysis [22]. The leucine metabolite beta-hydroxy- beta-methylbutyric acid (HMB) has also been well-described with regard to its effects on whole-body muscle mass accretion [23-25], as well as its ability to independently activate skeletal muscle mTOR signaling and reduce proteolytic signaling [26-28]. Creatine monohydrate has been less studied with regards to mTOR pathway modulation, although some evidence exists suggesting that creatine monohydrate is able to increase myotube differentiation through poorly understood mechanisms [29]. Notwithstanding, ample literature has demonstrated that creatine monohydrate supplementation is able to increase muscle mass and strength [30-35].

While a plethora of literature reports the effects of these nutritional supplements on skeletal muscle anabolic and/or anti-catabolic mechanisms, no information to our knowledge is known regarding how or if these supplements can abrogate facets of MSTN signaling. Therefore, the purpose of this study was to determine whether differentiated/mature myotubes treated with leucine, HMB or creatine monohydrate in the presence of MSTN affected: a) myotube diameter, b) select anabolic indices (Protein: DNA, RNA: DNA), and c) the mRNA expression patterns of genes associated with myostatin signaling.

## Methods

### Cell culture methods

C2C12 myoblasts (graciously donated by RHA), passage no. 4–10, were maintained in growth medium (GM; DMEM, 10% FBS, 1% penicillin/streptomycin, 0.1% gentamycin) under standard culture conditions at 37°C in a 5% CO_2_ atmosphere. Myoblasts were grown on 145 mm plates (Griener Bio-One GmbH, Maybachstr, Frickenhausen, GER) at a density of 7.5 × 10^5^ in 10 ml of growth medium for protein analyses, or on 12-well plates (Griener Bio-One GmbH) at a density of 5 × 10^5^ for mRNA analyses. Differentiation was induced 48 h after myoblast growth reached 80%–90% confluency by removing the growth medium and replacing it with differentiation medium (DM; DMEM, 2% (vol/vol) horse serum, 1% penicillin/streptomycin). DM was then replaced every 24 h for 4 d.

### Treatment methods (DM only, MSTN only, MSTN + Leucine, MSTN + HMB, MSTN + Creatine)

**Figure 1:**
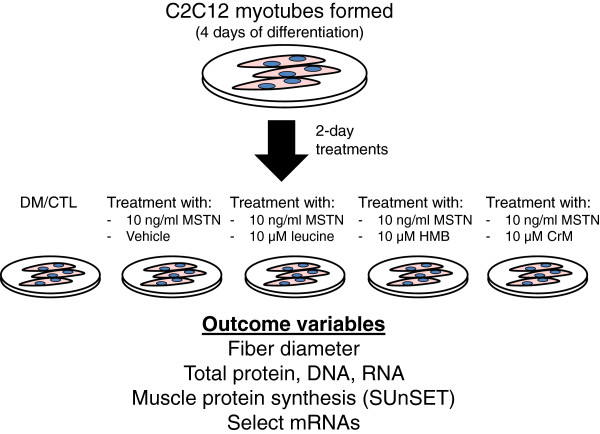
**Study design.** After four days of differentiation, cells were treated for two days (three times per day) with differentiation media (DM) and vehicle (10 mM Tris-NaCl), 10 ng/ml recombinant GDF-8 MSTN, 10 ng/ml MSTN and 10 μM leucine, 10 ng/ml MSTN and 10 μM of HMB, or 10 ng/ml MSTN and 10 μM creatine monohydrate.

The study design is illustrated in Figure 1 below. Briefly, after four days of differentiation, cells were treated three times per day with one of the following treatments for 48 h: 1) DM and vehicle (10 mM Tris-NaCl); denoted as ‘DM/CTL’ only, 2) 10 ng/ml rGDF-8 MSTN (R&D Systems, Minneapolis, MN, USA); denoted as ‘MSTN’, 3) 10 ng/ml MSTN and 10 μM (13 ug/ml) leucine (EMD Chemicals, Inc., San Diego, CA, USA); denoted as MSTN + Leu, 4) 10 ng/ml MSTN and 10 μM (13 ug/ml) of free acid HMB (Metabolic Technologies, Inc., Ames, IA, USA); denoted as ‘MSTN + HMB’, or 5) 10 ng/ml MSTN and 10 μM (12 ug/ml) creatine monohydrate (BodyBuilding.com, Boise, ID, USA); denoted as ‘MSTN + Crea’. The MSTN treatment dosage was based upon two prior studies showing that 10–30 ng/ml reduces myotube diameter in differentiated C2C12 myotubes [36,37]. The leucine, HMB and creatine monohydrate dosages were based upon prior C2C12 literature showing biological responses to similar dosages for each respective ingredient [21,38,39]. On the last day of treatment, and 30–45 min prior to cell lysis, cells were pulse-labeled with 1 μM of puromycin hydrochloride (Millipore, Temecula, CA, USA) in phosphate-buffered saline for subsequent muscle protein synthesis (MPS) assessment.

Cells grown on 145 mm plates were lysed using RIPA buffer (Tris base; pH 8.0, NaCl, NP-40, sodium deoxycholate, SDS) containing protease and phosphatase inhibitors (Ameresco, Solon, OH, USA). After cells were lysed, RIPA homogenates were analyzed for total protein, total DNA, and total RNA using a Qubit Fluorometer (Life Technologies, Grand Island, NY). RIPA homogenates were then spun down at 500xg for 5 min, and supernatants were stored for Western blotting analyses as described below.

### Light Microscopy Imaging and ImageJ Analysis

Prior to cell lysis of the 145 mm plates, 10× digital images were obtained for n = 2 for each treatment using light microscopy (Nikon Eclipse Ci-L) and digital capture (Nikon DS-QilMc). Fiber diameters from 150–190 myotubes per condition were obtained and assessed using ImageJ (NIH, Bethesda, MD, USA).

### Western blotting methods and analysis

In order to examine if MSTN treatment reduced muscle protein synthesis (MPS) rates compared to DM/CTL myotubes, the SUnSET method was employed [40]. Briefly, RIPA homogenates from 145 mm plates were subjected to 4-20% SDS-polyacrylamide gel electrophoresis using pre-casted gels (C.B.S. Scientific Company, San Diego, CA, USA). Proteins were transferred to polyvinylidene difluoride membranes (Whatman™, Westran® Clear Signal), and membranes were blocked for 1 h at room temperature with 5% nonfat milk powder. Mouse anti-puromycin (1:5,000; Millipore) was then incubated with membranes overnight at 4°C in 5% bovine serum albumin, and the following day membranes were incubated with anti-mouse IgG secondary antibodies (Cell Signaling, Danvers, MA, USA) at room temperature for 1 h. Membranes were then developed using an enhanced chemiluminescent reagent (Amersham, Pittsburgh, PA, USA), and band densitometry was performed through the use of a UVP Imager and associated densitometry software (UVP, LLC, Upland, CA, USA).

### RNA isolation and real-time PCR

RNA was isolated from myotubes grown on 12-well plates using Ribozol (Ameresco) per the manufacturer’s recommendations. 400 ng of cDNA was synthesized using a cDNA synthesis kit (Quanta, Gaithersburg, MD, USA) per the manufacturer’s recommendations. Real-time PCR was performed using mRNA specific primers {Akirin-1/Mighty: forward primer 5′- ATACAGTCACGGAGCTCCCT-3′, reverse primer 5′- ACTTGTTACACGCTCCGAGG-3′; Atrogin-1/MAFbx: forward primer 5′- CCATCCTCTTTCTTGCCCGT-3′, reverse primer 5′- ATCACTGTCCAACCTGGCTG-3′; MuRF-1: forward primer 5′- TGGGACAGATGAGGAGGAGG-3′, reverse primer 5′- TTTACCCTCTGTGGTCACGC-3′; beta-actin: forward primer 5′- GTGGATCAGCAAGCAGGAGT-3′, reverse primer 5′- ACGCAGCTCAGTAACAGTCC-3′; Notch-1: forward primer 5′- TGGACTGTTCTGTGCATCCC-3′, reverse primer 5′- TGGGGATCAGAGGCCACATA-3′; Ski: forward primer 5′- CCCACATGCCAGGATGACTT-3′, reverse primer 5′- GCTTTGCCAACTTCACCCAG-3′; MyoD: forward primer 5′- CCTGCCCTCCACATCCTTTT-3′, reverse primer 5′- GAAGGGGGAGAGTGGGGTAT-3′} and SYBR green chemistry (Quanta). Primer efficiency curves for all genes were generated and efficiencies ranged between 90% and 110%.

### shRNA experiments to confirm that Akirin-1/Mighty affects myotube size

A separate batch of C2C12 myoblasts was seeded in 12-well plates at a density of 5 × 10^5^ cells per plate. Cells were grown to 80-90% confluency and then transfection growth media containing Lipofectamine 3000 (Life Technologies) was added to myoblasts per the manufacturer’s recommendations. Specifically, n = 4–5 wells were transfected with green fluorescent protein (GFP) reporter plasmids (GeneCopoeia, Rockville, MD, USA) containing either: a) scrambled shRNA, or b) Akirin-1/Mighty shRNA. Twenty-four hours after transfection, cells were differentiated as mentioned above. Cells were then allowed to differentiate for 4 days prior to imaging on an inverted fluorescent microscope (Olympus IX71). 10× digital fluoromteric FITC-filtered images were obtained for each transfection condition and myotube areas of GFP-positive myotubes were quantified using ImageJ. Cells were then lysed with Ribozol (Ameresco) per the manufacturer’s recommendations and Akirin-1/Mighty mRNA knock-down was confirmed using real-time PCR methods as mentioned above.

### Statistics

All data are presented as means ± standard error. For all data, statistics were performed between treatments using an ANOVA with LSD post-hoc comparisons when applicable. All statistics were performed using IBM SPSS version 22.0 and significance was determined at p < 0.05.

## Results

### L-leucine, HMB and creatine monohydrate prevent myostatin-induced myotube atrophy

**Figure 2:**
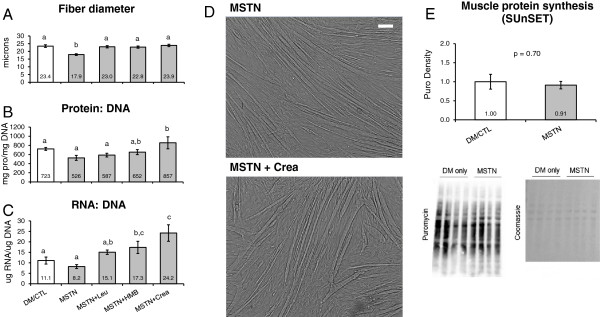
**Efects of MSTN in the absence or presence of leucine, HMB, or creatine on markers of skeletal muscfle hypertrophy.** Legend: Data are presented as means ± standard error. Data in **A** show that MSTN treatment decreases myotube diameter, while Leu, HMB, and Crea rescue MSTN-induced atrophy. Data in **B & C** are n = 5-6 plates per treatment. Muscle protein synthesis data in **E** are n = 4 treatments per plate; of note, MSTN+Leu, MSTN+HMB and MSTN+Crea samples were separately analyzed for MPS (data not shown). One-way ANOVA with LSD post-hoc comparisons performed where applicable; difference superscript letters = p < 0.05 and NS = no significant differences. In subfigure **D** of 10x light micrographs, white bar is 100 μm.

Two days of MSTN only treatment significantly reduced myotube diameter by approximately 30% compared to the DM/CTL condition (p < 0.001; Figure 2A). However, the MSTN + Leu, MSTN + HMB and MSTN + Crea treatments rescued this atrophy effect. MSTN treatment tended to decrease the protein: DNA compared to the DM/CTL condition (p = 0.084, Figure 2B; index of muscle hypertrophy [41]). However, MSTN + Crea myotubes exhibited a significantly greater protein: DNA ratio compared to the MSTN only treatment (p < 0.01, Figure 2B). MSTN + Leu, MSTN + HMB, and MSTN + Crea myotubes all exhibited a greater RNA: DNA ratio compared to the MSTN only condition (Figure 2C; index of translational capacity and hypertrophic potential [41]).

Interestingly, two days of MSTN treatment did not affect MPS rates compared to DM/CTL only-treated myotubes (Figure 2E). Thus, the MSTN-induced reduction in myotube diameter appears to be independent of MPS rates. Notably, MPS rates were also determined in a separate batch of DM/CTL (n = 2), MSTN + Leu (n = 6), MSTN + HMB (n = 6), and MSTN + Crea (n = 6) myotubes. Compared to DM/CTL myotubes, MSTN + Leu and MSTN + HMB caused non-significant increases in MPS rates (38% and 31%, respectively; *data not shown*), whereas MSTN + Crea did not increase MPS levels.

### Myostatin-induced down-regulation of Akirin-1/Mighty mRNA is rescued by creatine treatments

**Figure 3:**
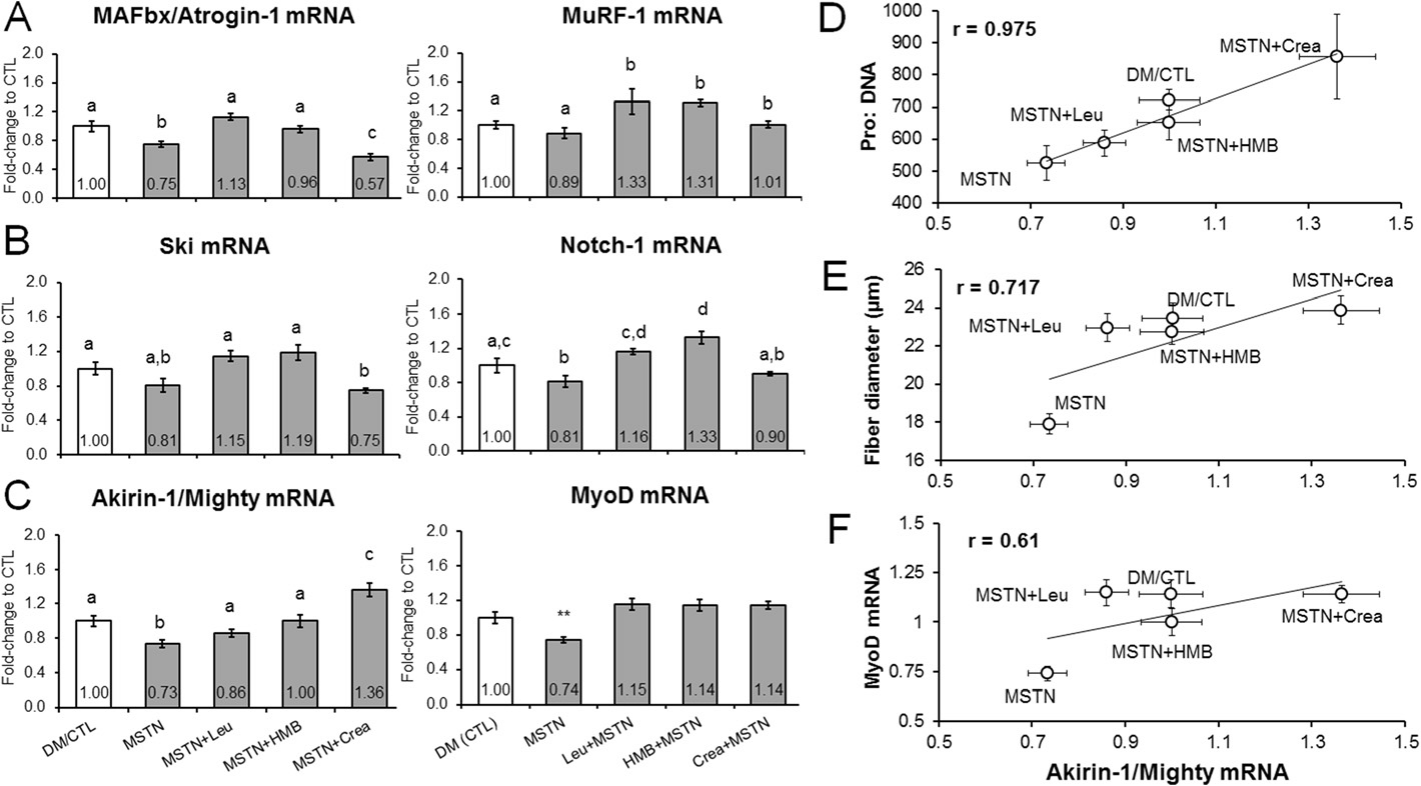
**Effects of MSTN in the absence or presence of leucine, HMB, or creatine on the expression of MSTN-related mRNAs.** Legend: Data are presented as means ± standard error (n = 6–9 plates per treatment). Effects of MSTN with or without each ingredient on the mRNA expression of explicit atrogenes **(A)**, MSTN signaling repressors **(B)**, and MSTN transcriptional targets **(C)**. Sub-figures **D** and **E** demonstrate that Akirin-1/Mighty mRNA expression was modestly-to-strongly correlated with measured hypertrophy variables. Sub-figure **F** shows that Akirin-1/Mighty mRNA expression was modestly correlated with the early differentiation marker MyoD mRNA. One-way ANOVA with LSD post-hoc comparisons performed in sub-figures **A**/**B**/**C**; difference superscript letters = p < 0.05. In sub-figure **C**, ** indicates that MSTN down-regulated MyoD mRNA compared to all other groups (p < 0.01).

Given that MSTN-treated myotubes atrophied independent of MPS rates, we next examined if select atrogenes (MAFbx/atrogin-1, MuRF-1), MSTN signaling repressors (Notch-1, Ski), and/or MSTN transcriptional targets (Akirin-1/Mighty, MyoD) were affected at the mRNA level by MSTN treatment with or without leucine, HMB or creatine monohydrate treatments. Compared to DM/CTL myotubes, the MSTN-only treatment did not affect the mRNA expression patterns of MuRF-1, Ski, Notch-1. However, MAFbx/atrogin-1 mRNA levels were depressed compared to DM/CTL myotubes (Figure 3A/B).The MSTN-only treatment did depress Akirin-1/Mighty mRNA and MyoD mRNA levels by 27% (p < 0.001) and 26% (p < 0.01) compared to DM/CTL myotubes (Figure 3C). Moreover, MSTN-treated myotubes co-treated with leucine or HMB reversed MSTN-induced Akirin-1/Mighty mRNA down-regulation (p < 0.05); specifically, MSTN + Leu exhibited an 18% increase and MSTN + HMB exhibited a 27% increase in Akirin-1/Mighty mRNA compared to MSTN-treated myotubes. MSTN + Crea-treated myotubes exhibited a significant up-regulation in Akirin-1/Mighty mRNA levels by 36% compared to DM/CTL myotubes (p < 0.05) and 86% compared to MSTN-only treated myotubes (p < 0.001). The expression of Akirin-1/Mighty mRNA was modestly (0.50 < r < 0.80) to strongly (r > 0.80) correlated with select measured anabolic indices (Pro:DNA r = 0.98, p = 0.004; fiber diameter r = 0.72, p > 0.05; Figure 3D/E) as well as the early differentiation marker MyoD mRNA (r = 0.61, p > 0.05; Figure 3F).

### Knockdown of Akirin-1/Mighty mRNA decreases myotube size

**Figure 4:**
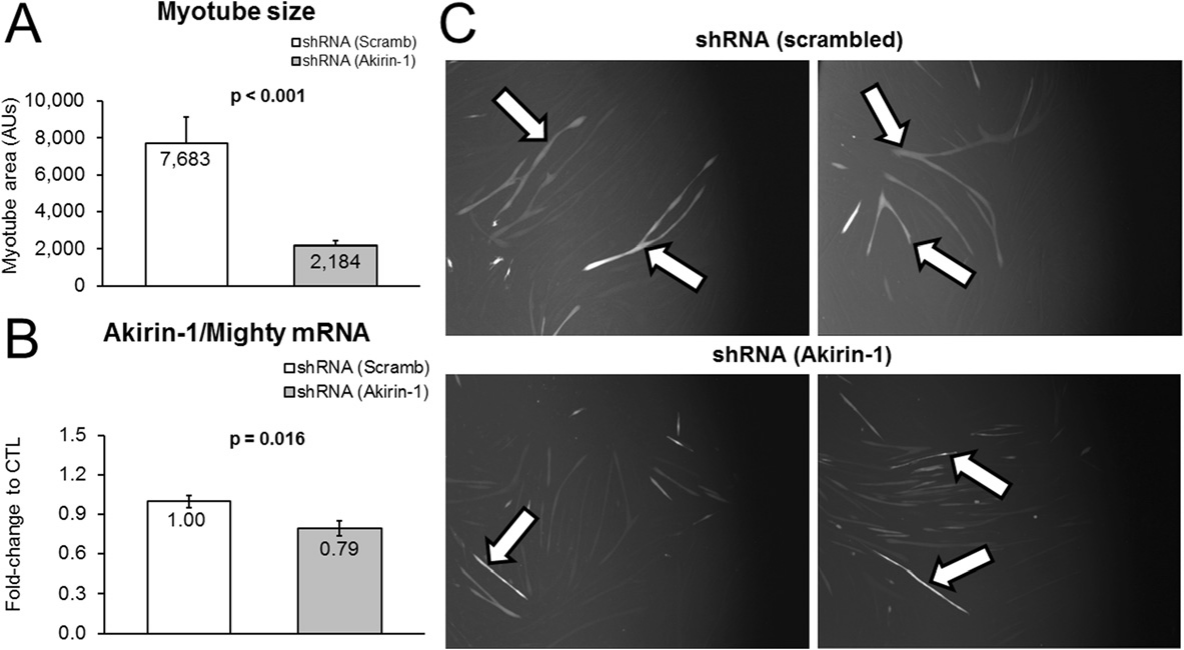
**Experiment demonstrating Akirin-1/Mighty mRNA knockdown affects myotube size.** Legend: Data are presented as means ± standard error (n = 4–5 plates per treatment). Effects of shRNA-mediated Akirin-1/Mighty mRNA knockdown on myotube size versus a scrambled shRNA control condition **(A)**, and confirmation that Akirin-1 mRNA was reduced in shRNA-transfected myotubes **(B)**. Photographs in sub-figure **C** are 10x representative images of GFP-positive cells (arrows) that were transfected with either a scrambled (CTL)-shRNA or Akirin-1/Mighty-shRNA.

As mentioned above, Akirin-1/Mighty mRNA expression patterns between treatments exhibited modest to strong correlations to select hypertrophy indices. Thus, we next sought to determine if experimentally decreasing Akirin-1/Mighty mRNA using shRNA knockdown affected myotube size. Indeed, GFP-positive myotubes transfected with the Akirin-1/Mighty shRNA plasmid exhibited a drastic reduction in myotube area (−72%, p < 0.001; Figure 4A). Knockdown in transfected wells was also confirmed at the mRNA level (Figure 4B). Therefore, we posit that MSTN-induced atrophy observed in the current study is likely linked to the MSTN-induced down-regulation in Akirin-1/Mighty mRNA. Furthermore, leucine and HMB appear to prevent this down-regulation and creatine monohydrate increases myotube Akirin-1/Mighty mRNA levels in spite of MSTN treatment.

## Discussion

We used an *in vitro* approach to investigate whether leucine, HMB, or creatine monohydrate could rescue the atrophic effects of MSTN in C2C12 myotubes. MSTN has previously been shown to inhibit myoblast proliferation, myotube differentiation and protein synthesis in the C2C12 cell line [42-44]. Specifically, one of the aforementioned studies demonstrated that myotubes treated with high doses of recombinant MSTN (~1-6 μg/ml which is 100-600x the dose used in the current study) significantly depressed protein synthesis in myotubes [42]. However, the relatively low concentration of MSTN treatments used in the current study reduced myotube size independent of muscle protein synthesis. This finding is in agreement with other studies which used MSTN treatment dosages similar to our study (10–30 ng/ml) and reported MSTN to reduce myotube diameter by inhibiting differentiation in C2C12 myotubes [36,37]. Thus, we hypothesize that MSTN-induced atrophy in the current study was likely due to diminished myotube differentiation rather than decreases in muscle protein synthesis and/or increases in muscle proteolysis; a hypothesis which is further supported by the MSTN-induced down-regulation in MyoD mRNA.

Interestingly, while myotubes treated with MSTN only showed a significant decrease in myotube diameter, this effect was reversed in all three treatment groups (MSTN + Leu, MSTN + HMB, and MSTN + Crea). There is ample evidence to suggest that leucine and HMB are able to increase muscle protein synthesis *in vitro* and *in vivo*[26,27,45,46]. Thus, it is difficult to reconcile why MSTN + Leu and MSTN + HMB treatments in the current study did not statistically increase muscle protein synthesis compared to DM/CTL myotubes. However, all treatments occurred in myotubes that were not amino acid deprived; this being a condition which may be obligatory for leucine and HMB to exert more profound muscle protein synthesis effects [47]. Furthermore, the MSTN + Crea treated group demonstrated the greatest hypertrophic effect in spite of the fact that creatine monohydrate likely does not affect markers of muscle protein synthesis [29] and/or muscle protein synthesis rates [48]*in vivo*. Thus, we hypothesized that leucine, HMB and creatine monohydrate treatments all independently counteracted low-dose MSTN-induced atrophy through potential genetic mechanisms related to myotube differentiation.

Of the mRNAs measured in the current study select hypertrophic indices, including the protein: DNA and myofiber diameter, were strongly and modestly correlated with Akirin-1/Mighty gene expression, respectively. Furthermore, our main findings with Akirin-1/Mighty gene expression were as follows: 1) leucine and HMB can reverse MSTN-induced down-regulation in Akirin-1/Mighty mRNA; 2) in spite of MSTN treatment, creatine monohydrate up-regulated Akirin-1/Mighty mRNA while exhibiting the most potent anabolic effects; and 3) Akrin-1/Mighty mRNA knockdown via shRNA transfection reduced myofiber size. Hence, our findings support the hypothesis that the transcriptional modulation of Akirin-1/Mighty mRNA by leucine/HMB/creatine monohydrate may be a mechanism whereby these ingredients promote myotube hypertrophy *in vitro* in spite of MSTN treatments.

Our finding that each of these purported anabolic ingredients rescues MSTN-induced Akirin-1/Mighty mRNA down-regulation is indeed difficult to interpret from a mechanistic and practical viewpoint regarding the preservation of myofiber size. Furthermore, this is the first study to show that each of these ingredients affects (directly or indirectly) the mRNA expression of Akirin-1/Mighty mRNA. Resistance exercise has been shown to increase Akirin-1/Mighty mRNA expression patterns 6 h and 48 h following an acute exercise bout in rodents [49]; of note these rodents were trained 6 weeks prior to the acute bout and the exercise-induced changes in Akrin-1/Mighty strongly predicted the hypertrophic response to the 6-week training bout. The authors concluded that, while Akrin-1/Mighty may play a role in the activation of satellite cells, how Akirin-1/Mighty promotes the hypertrophic response to exercise has yet to be determined. Notwithstanding, it appears that Akirin-1/Mighty plays a role in exercise-induced skeletal muscle hypertrophy. The expression of mRNA expression of akirin genes in skeletal muscle are sensitive to fasting and re-feeding in Artic charr [50], and we have observed mixed gastrocnemius Akirin-1/Mighty mRNA to increase approximately 90% 3 h after rats fed 10 human equivalent grams of whey protein concentrate (which is ~12-14% leucine) when compared to 18-h fasted rats (p < 0.05; *unpublished observations*). Hence, our finding that purported anabolic ingredients directly or indirectly affect myotube Akirin-1/Mighty mRNA expression is not unfounded given that other hypertrophic stimuli (i.e., resistance exercise and protein feeding) have also been shown to increase the expression of this gene in skeletal muscle. In this regard, future research is needed to elucidate if: 1) decrements in Akirin-1/Mighty mRNA expression accompany and/or causal to muscle wasting conditions such as sarcopenia, cachexia, and disuse atrophy; and 2) if each of the anabolic ingredients studied herein mitigate these conditions through Akirin-1/Mighty gene expression changes.

While the exact mechanisms are unknown as to how Akirin-1/Mighty regulates muscle mass, it is a proven transcriptional target of myostatin [49,51,52]. Recent evidence also suggests that the Akirin-1/Mighty gene modulates satellite cell proliferation and differentiation following muscle injury, and there is interplay between Akirin-1/Mighty down-regulation and the inhibition of differentiation-promoting genes such as MyoD [53]. There is a paucity of literature examining how leucine and/or HMB affect Akirin-1/Mighty mRNA expression patterns, though recent *in vivo* evidence suggests that chronically consuming a leucine-rich pre-exercise beverage increases skeletal muscle MyoD and MRF4 mRNA [54]. Furthermore, adding various concentrations (10–100 μg/ml) of HMB to serum-starved myoblasts has been shown to induce myoblast proliferation and MyoD expression, suggestive of enhanced myoblast differentiation [55]. However, Akirin-1/Mighty mRNA was not assessed in the aforementioned studies making it difficult to reconcile whether these effects were mitigated through Akrin-1/Mighty mRNA gene expression.

Of particular interest was the ability of creatine monohydrate to promote the up-regulation of Akirin-1/Mighty mRNA in the presence of myostatin. Creatine monohydrate supplementation has been shown to reduce the catabolic response of hind limb immobilization in rats [56]. Additionally, Johnston et al. [57] have demonstrated that short-term creatine monohydrate supplementation (7 days) attenuates losses of muscle mass and strength during upper-arm immobilization in young men. Furthermore, prolonged creatine monohydrate supplementation has been reported to increase satellite cell proliferation and differentiation in resistance-trained subjects versus a placebo group [58]. While the anabolic/anti-catabolic mechanisms of creatine monohydrate remain poorly understood, creatine monohydrate supplementation has been shown to increase cellular fluid retention and modulate the expression of myogenic transcription factors related to skeletal muscle hypertrophy [59,60]. With regards to the former mechanism, Häussinger [61] reported that an increase in cellular fluid/swelling acts as an anabolic proliferative signal, whereas cell shrinkage is catabolic and anti-proliferative. However, creatine monohydrate has been shown to promote myotube hypertrophy *in vitro* by enhancing myotube differentiation compared to DM/CTL-treated myotubes [38]; an effect the authors suggested may be independent of the intracellular osmolarity effects of creatine monohydrate. Therefore, the ability of creatine monohydrate to up-regulate Akirin-1/Mighty mRNA may be a primary mechanism involved in the ability of creatine monohydrate to stimulate skeletal muscle hypertrophy independent of muscle protein synthesis and/or its effects on osmolarity. In this regard, future research should continue to examine the ability of creatine monohydrate supplementation to modulate Akirin-1/Mighty mRNA expression *in vivo*.

## Conclusions

We demonstrate that leucine, HMB, and creatine monohydrate reverse myostatin-induced atrophy in myotubes; this potentially results from the independent action of each ingredient modulating Akirin-1/Mighty mRNA expression. Furthermore, our findings suggest that, in spite of MSTN treatments, creatine monohydrate treatment up-regulates Akirin-1/Mighty mRNA which leads to a hypertrophic effect clearly independent of muscle protein synthesis. Future *in vivo* studies should continue to examine how leucine, HMB, and/or creatine monohydrate independently or synergistically affect Akirin-1/Mighty gene expression. More importantly, while Akirin-1/Mighty gene expression is needed for the maintenance of myofiber size as reported herein, further research is needed in order to examine how Akirin-1/Mighty gene expression mechanistically relates to skeletal muscle hypertrophy *in vivo*.

## Abbreviations

HMB: Beta-hydroxy-beta-methylbutyric acid; Leu: L-leucine; Crea: Creatine monohydrate; MSTN: Myostatin; DM: Differentiation media; CTL: Control; SUnSET: Surface sensing of translation.

## Competing interests

Besides SB, and JAR, none of the authors have non-finacial and/or financial competing interests. SB, and JAR are employed by Metabolic Technologies, Inc., but both authors intellectually contributed to study design and data write-up. Therefore, all co-authors agreed that their intellectual input into this project warranted co-authorship.

## Authors’ contributions

CDF, BSF, RHA, VJD, SB, JAR, JMW, MDR. This person has made substantial contributions to conception and design, or acquisition of data, or analysis and interpretation of data. CBM, MDR. This person primarily was involved in drafting the manuscript or revising it critically for important intellectual content. CBM, CDF, BSF, RHA, VJD, SB, JAR, JMW, MDR. This person gave final approval of the version to be published. CBM, CDF, BSF, RHA, VJD, SB, JAR, JMW, MDR This person agrees to be accountable for all aspects of the work in ensuring that questions related to the accuracy or integrity of any part of the work are appropriately investigated and resolved.
